# Mechanical Ventilation in Neonatal Respiratory Distress Syndrome at High Altitude: A Retrospective Study From Tibet

**DOI:** 10.3389/fped.2019.00476

**Published:** 2019-11-19

**Authors:** Dan Chen, Xiuxiu Liu, Jiujun Li

**Affiliations:** ^1^Department of Neonatology, Shengjing Hospital of China Medical University, Shenyang, China; ^2^Department of Pediatrics, Naqu People's Hospital, Naqu, China; ^3^Plateau Medical Research Center of China Medical University, Paediatric Intensive Care Unit, Shengjing Hospital of China Medical University, Shenyang, China

**Keywords:** high altitude, neonate, respiratory distress syndrome, mechanical ventilation, arterial partial pressure of oxygen

## Abstract

**Objective:** To explore the characteristics of mechanical ventilation parameters and the arterial partial pressure of oxygen in neonatal respiratory distress syndrome (RDS) at high altitude.

**Methods:** From the 1st May 2017 to the 31st December 2018, we recruited 33 neonates with severe RDS who were undergoing mechanical ventilation in the NICU of Naqu People's Hospital in Tibet (4,580 m above sea level); these neonates formed a plateau observation group. We also recruited a non-plateau control group: 66 neonates with severe RDS undergoing mechanical ventilation of Shengjing Hospital in Liaoning (51 m above sea level). Various ventilation parameters and the arterial partial pressure of oxygen were then compared between the two groups, between the survivors of the two groups, and between those who died and survived in the plateau group.

**Results:** In terms of initial ventilator parameters, peak inspiratory pressure (PIP), positive end expiratory pressure (PEEP), and the fraction of inspired oxygen (FiO2) in the plateau group were significantly higher than those in the non-plateau group (*P* < 0.01). PIP, PEEP, and FiO2 in the survivors from the plateau group were also significantly higher than those in the non-plateau group (*P* < 0.01). In addition, the arterial partial pressure of oxygen in the non-plateau group was higher (*P* < 0.05) than that in the plateau group during the early postnatal period, and the arterial partial pressure of oxygen at 6 and 12 h was lower than that in the plateau group (*P* < 0.05).

**Conclusion:** Mechanical ventilation can effectively improve the arterial oxygen partial pressure and reduce the mortality of newborns with RDS in a plateau environment. It was clearly evident that ventilation parameters are closely related to altitude. It is therefore not advisable to apply mechanical ventilation parameters used in a non-plateau area as a guide for the treatment of newborns with RDS in plateau areas.

## Introduction

Approximately 140 million people around the world, who live at an altitude higher than 2,438 meters, are at high risk of hypoxia (1). Tibet, with a mean altitude of 4,000 meters, is the highest geographical region in China and Asia. In Tibet, the atmospheric pressure, measured at 3,600 meters above sea level, is <500 mmHg, while the partial pressure of oxygen during inhalation is ~100 mmHg (2). Some previous studies have suggested that with increasing altitude, the partial pressure of inhaled oxygen also decreases, and that this leads to an increased incidence of neonatal mortality and intrauterine growth restriction (3–6). In one previous study, Moore et al. found that the mortality rate of newborns with neonatal respiratory distress syndrome (RDS) was negatively correlated with altitude (7). One possible factor for this is that air pollution is a problem in low altitude areas (8, 9). RDS is a common problem in newborns, particularly in premature infants. Thus far, only a few studies have investigated RDS in high altitude areas (7, 10–12). Most of these previous studies have suggested that the incidence of RDS is independent of altitude, although the use of pulmonarysurfactant (PS) is greater in high altitude areas (10, 12). The mean altitude of the Naqu area in Tibet is 4,500 meters, the atmospheric pressure and oxygen content in this area is half that of low altitude plains. From May 2017, medical institutions in the Naqu area began using mechanical ventilation, the mortality rate of infants with severe symptoms decreased significantly. However, some neonates, particularly those with RDS, died due to failure of treatment. Until now, there is no published studies which have observed and evaluated the use of mechanical ventilation in neonates with RDS in high altitude areas. The present study aimed to explore the characteristics of mechanical ventilation conditions and the arterial partial pressure of oxygen in high altitude areas by analyzing neonates with severe RDS undergoing mechanical ventilation in the Naqu area of Tibet.

## Methods and Materials

### Subjects

Between the 1st May 2017 and the 31st December 2018, we recruited 33 neonates with severe RDS who were undergoing mechanical ventilation in the NICU of Naqu People's Hospital of Tibet (4,580 m above sea level), these formed our plateau observation group. We also recruited 66 neonates with severe RDS who were undergoing mechanical ventilation in Shengjing Hospital of Liaoning (51 m above sea level) as a non-plateau control group.

Our specific inclusion criteria were as follows: (1) RDS neonates hospitalized and requiring mechanical ventilation immediately after birth [Diagnostic criteria for RDS: (a) acute onset, shortly after birth, respiratory distress manifesting as shortness of breath, cyanosis, three concave sign upon inhalation, oxygen ineffective, requirement for auxiliary ventilation; (b) progressive respiratory distress; (c) chest X-ray film showing a general reduction in the translucency of both lungs, reticular, granular shadows, bronchial bronchograms, and severe cases showing “white lungs;” (d) except for other diseases causing respiratory distress]; (2) Synchronized intermittent mandatory ventilation (SIMV) mode was adopted during mechanical ventilation; (3) Chest X-rays were consistent with severe RDS changes, including ground glass opacity accompanied by bronchial inflation, blurred cardiac boundary or “white lungs;” and (4) pulmonarysurfactant (PS) was either used only once or not at all.

Our exclusion criteria were as follows: (1) Neonates with congenital malformations or hereditary metabolic diseases and (2) Newborns with severe asphyxia, infection or other diseases that may cause death.

### Data Collection

For each subject, ~1 ml of radial artery blood was collected within 30 min (immediately after mechanical ventilation and prior to the use of PS), 1, 6, and 12 h after birth. The partial pressure of carbon dioxide and oxygen in the arterial blood was measured using a blood gas analyzer.

A range of clinical data were recorded, including gestational age, gender, birth weight, ethnicity, 1 and 5 min Apgar scores, the application of PS; the arterial partial pressure of carbon dioxide and the arterial partial pressure of oxygen within 30 min, 1, 6, and 12 h after birth; positive end-inspiratory pressure (PIP), positive end-expiratory pressure (PEEP), and the fraction of inspired oxygen (FiO2) prior to the use of PS; duration of mechanical ventilation and outcome.

### Instruments and Equipment

In the plateau group, we used the Mindray SV600 ventilator (Shenzhen, China; altitude was set to 4,500 meters) and the Radiometer ABL80 blood gas analyzer (Bronshoj, Denmark). In the non-plateau group, we used the Babylog VN500 ventilator (Lubeck, Germany) and the Roche Cobas B 123 blood gas analyzer (Rotkreuz, Switzerland).

### Statistical Analysis

SPSS 13.0 (SPSS, Chicago, IL, USA) was used for the processing and statistical analysis of data. Data are presented as means ± standard deviation (SD). Statistical analysis involved the student's *t*-test, the χ^2^ test, and Fisher's exact test. *P* < 0.05 was considered to indicate a statistically significant difference.

## Results

### General Information

The plateau group featured 33 Tibetan neonates with RDS; 18 of these used porcine PS (240 mg regardless of weight) within 5 to 12 h of birth and 17 subjects died. There were 66 neonates in the non-plateau group, all were of Han nationality; 65 of the neonates used porcine PS (200 mg/kg) within 1 h of birth and one subject died. Respiratory failure was the cause of death in both groups.

### Initial Mechanical Ventilation Parameters and the Partial Pressure of Oxygen at Different Time Points in Neonates With RDS at High Altitude

**Figure 1:**
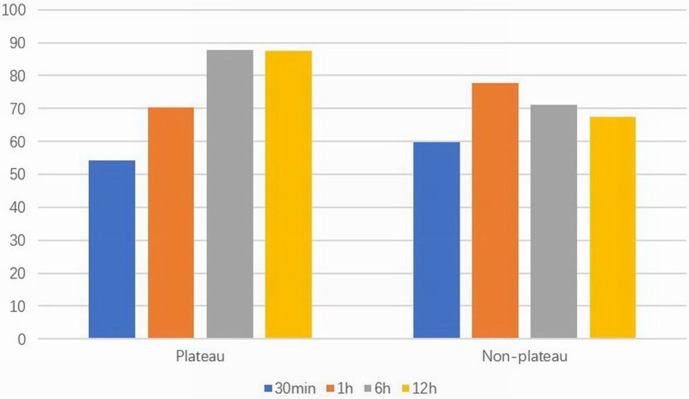
Arterial partial pressure of oxygen (mmHg) at different time points in two groups.

Birth weight in the plateau group was significantly higher than that of the non-plateau group (*P* < 0.05), while the utilization rate of PS was significantly higher in the non-plateau group (*P* < 0.01). Blood gas analysis showed that the arterial partial pressure of oxygen within 30 min and 1 h after birth in the plateau group was lower than that in the non-plateau group, while the arterial partial pressure of oxygen at 6 and 12 h after birth was significantly higher than that in the non-plateau group (*P* < 0.05). The partial pressure of carbon dioxide 12 h after birth was significantly higher than that in the non-plateau group (*P* < 0.01), although there were no statistically significant differences when compared between the two groups at the 30 min, 1, and 6 h timepoints. Initial ventilation parameters (PIP, PEEP, and FiO2) and mortality following postnatal mechanical ventilation were significantly higher in the plateau group (*P* < 0.01). Please refer to Figure 1 and Table 1.

**Table 1:** Comparison of clinical data between neonates with RDS at plateau group and non-plateau group.

	**Plateau**	**Non-plateau**	***t*/χ^**2**^-value**	***P-*value**
	***n* = 33**	***n* = 66**		
**MEDICAL HISTORY DATA**				
Gestational age (weeks)	30.5 ± 2.2	30.4 ± 2.2	0.235	0.815
Gender (male/female)	24/9	50/16	0.107	0.744
Birthweight (g)	1681.8 ± 324.5	1490.4 ± 455.5	2.154	0.034
PS usage	18 (33)	65 (66)	31.348	<0.001
**BLOOD GAS ANALYSIS**				
**Within 30 min after birth**				
PaCO2	39.32 ± 18.04	44.64 ± 10.79	−1.560	0.126
PaO2	54.21 ± 8.14	59.74 ± 18.45	−2.064	0.042
**1 h after birth**				
PaCO2	39.56 ± 14.38	41.19 ± 9.03	−0.596	0.554
PaO2	70.42 ± 12.83	77.81 ± 22.62	−2.068	0.041
**6 h after birth**				
PaCO2	39.07 ± 7.78	35.61 ± 32.80	1.629	0.107
PaO2	87.75 ± 16.41	71.02 ± 22.85	4.139	<0.001
**12 h after birth**				
PaCO2	41.48 ± 8.89	33.32 ± 9.35	4.068	<0.001
PaO2	87.61 ± 16.04	67.40 ± 19.19	5.086	<0.001
**INITIAL PARAMETERS OF MECHANICAL VENTILATION**				
PIP	27.7 ± 4.4	21.4 ± 1.8	7.967	<0.001
PEEP	8.0 ± 2.4	5.4 ± 0.5	6.101	<0.001
FiO2	0.88 ± 0.13	0.47 ± 0.14	13.645	<0.001
**MORTALITY (%)**	51.5	1.5	36.972	<0.001

*PIP, peak inspiratory pressure; PEEP, positive end expiratory pressure; FiO2, fraction of inspired oxygen; PS, pulmonarysurfactant*.

### Mechanical Ventilation Parameters and Blood Gas Analysis at Different Time Points in Surviving RDS Neonates at Plateau Compared to Non-plateau

Compared with the non-plateau group, the utilization rate of PS was significantly lower in the plateau group (*P* < 0.01); there was no significant difference between the two groups in terms of gestational age, birth weight, or gender. The arterial partial pressure of oxygen within 30 min and 1 h after birth in the plateau group was significantly lower than that in the non-plateau group, while the arterial partial pressure of oxygen at 6 and 12 h after birth was significantly higher than that in the non-plateau group (*P* < 0.05). The partial pressure of carbon dioxide at 6 and 12 h after birth was significantly higher than that in the non-plateau group (*P* < 0.01).

**Figure 2:**
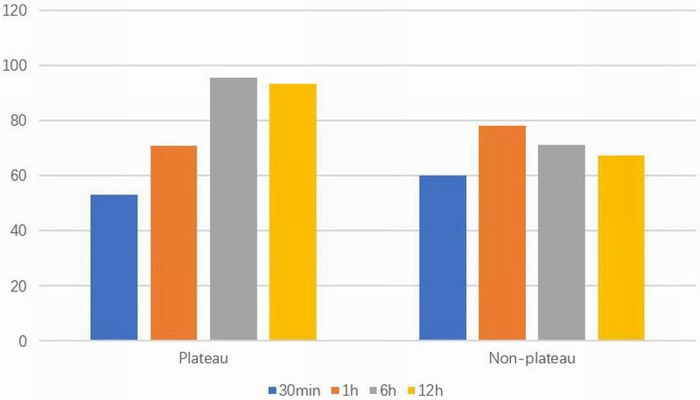
Arterial partial pressure of oxygen (mmHg) at different time points in two surviving groups.

The initial PIP and FiO2 in the plateau group were significantly higher than those in the non-plateau group (*P* < 0.01), although there were no significant statistical differences in the initial PEEP setting or mechanical ventilation time between the two groups. Please refer to Figure 2 and Table 2.

**Table 2:** A comparison of clinical data in surviving RDS neonates between plateau group and non-plateau group.

	**Plateau**	**Non-plateau**	***t*/χ^**2**^-value**	***P-*value**
	***n* = 33**	***n* = 66**		
**MEDICAL HISTORY DATA**				
Gestational age (weeks)	30.7 ± 2.2	30.4 ± 2.2	0.597	0.552
Gender (male/female)	11/5	49/16		0.751#
Birthweight (g)	1668.8 ± 33	1490.0 ± 459.1	1.464	0.147
PS usage	10 (16)	64 (65)		<0.001#
**BLOOD GAS ANALYSIS**				
**Within 30 min after birth**				
PaCO2	37.63 ± 16.93	44.48 ± 10.79	−1.543	0.140
PaO2	53.06 ± 7.18	59.84 ± 18.57	−2.322	0.023
**1 h after birth**				
PaCO2	38.06 ± 15.17	40.94 ± 8.85	−0.729	0.475
PaO2	70.88 ± 13.39	77.99 ± 22.75	−1.197	0.235
**6 h after birth**				
PaCO2	41.91 ± 8.76	34.88 ± 9.00	2.813	0.006
PaO2	95.56 ± 17.88	71.25 ± 22.95	4.587	<0.001
**12 h after birth**				
PaCO2	40.07 ± 9.55	32.92 ± 8.82	2.860	0.005
PaO2	93.13 ± 17.96	67.29 ± 19.32	4.855	<0.001
**INITIAL PARAMETERS OF MECHANICAL VENTILATION**				
PIP	24.5 ± 2.5	21.3 ± 1.7	6.201	<0.001
PEEP	6.1 ± 1.5	5.4 ± 0.5	1.827	0.087
FiO2	0.78 ± 0.10	0.47 ± 0.15	7.900	<0.001
**DURATION OF MECHANICAL VENTILATION (h)**	89.4 ± 47.7	61.0 ± 53.8	1.931	0.057

*#Fisher's exact test*.

*PIP, peak inspiratory pressure; PEEP, positive end expiratory pressure; FiO2, fraction of inspired oxygen; PS, pulmonarysurfactant*.

### A Comparison of Mechanical Ventilation Parameters and Blood Gas Analysis at Different Time Points in Neonates With RDS at High Altitude Between Those Who Survived and Those That Died

**Figure 3:**
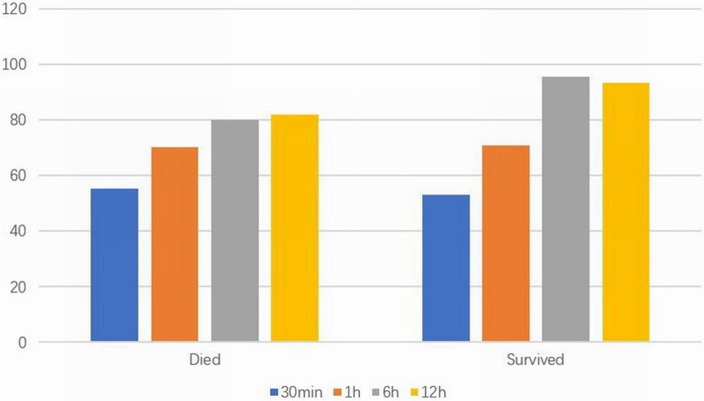
Arterial partial pressure of oxygen (mmHg) at different time points in died and survived groups at high altitude.

In the plateau group, there were no significant differences in terms of gestational age, sex, birth weight, cesarean section rate, Apgar score, and PS usage when compared between the neonates with RDS who died and those that survived. The partial pressure of arterial oxygen at 6 and 12 h in the group of neonates that died was significantly lower than that in the survival group (*P* < 0.05). The partial pressure of carbon dioxide at 6 h after birth was also significantly lower in the neonates that died compared to that in the surviving neonates (*P* < 0.05). Furthermore, the initial parameters of mechanical ventilation (PIP, PEEP, and FiO2) were also significantly higher in the group of neonates that died (*P* < 0.01). Please refer to Figure 3 and Table 3.

**Table 3:** A comparison of clinical data in neonates with RDS at high altitude between those that survived and those that died.

	**Died**	**Survived**	***t*/χ^**2**^-value**	***P-*value**
	***n* = 17**	***n* = 16**		
**MEDICAL HISTORY DATA**				
Gestational age (weeks)	30.2 ± 2.2	30.7 ± 2.2	−0.659	0.515
Gender (male/female)	13/4	11/5		0.708
Birthweight (g)	1694.1 ± 328.8	1668.8 ± 330.1	0.221	0.826
**Apgar score**				
1 min	4.59 ± 2.06	4.44 ± 1.79	0.224	0.824
5 min	7.29 ± 1.93	7.44 ± 1.03	−0.264	0.794
PS usage	8 (17)	10 (16)	0.793	0.373
**BLOOD GAS ANALYSIS**				
**Within 30 min after birth**				
PaCO2	40.92 ± 19.41	37.63 ± 16.93	0.519	0.608
PaO2	55.29 ± 9.04	53.06 ± 7.18	0.782	0.440
**1 h after birth**				
PaCO2	40.98 ± 13.91	38.06 ± 15.17	0.577	0.568
PaO2	70.00 ± 12.67	70.88 ± 13.39	−0.193	0.848
**6 h after birth**				
PaCO2	36.23 ± 5.59	41.91 ± 8.76	−2.185	0.037
PaO2	79.94 ± 10.33	95.56 ± 17.88	−3.027	0.005
**12 h after birth**				
PaCO2	42.99 ± 8.17	40.07 ± 9.55	0.911	0.370
PaO2	81.73 ± 11.57	93.13 ± 17.96	−2.084	0.046
**INITIAL PARAMETERS OF MECHANICAL VENTILATION**				
PIP	30.7 ± 3.6	24.5 ± 2.5	5.674	<0.001
PEEP	9.8 ± 1.6	6.1 ± 1.5	6.818	<0.001
FiO2	0.97 ± 0.06	0.78 ± 0.10	6.540	<0.001

*PIP, peak inspiratory pressure; PEEP, positive end expiratory pressure; FiO2, fraction of inspired oxygen; PS, pulmonarysurfactant*.

## Discussion

Our current data showed that the arterial partial pressure of oxygen during the early postnatal period of neonates with RDS requiring mechanical ventilation was significantly lower in the plateau area, the initial pressure and oxygen concentration settings were significantly higher than in the non-plateau area, and the mortality rate was also significantly higher than in the non-plateau area. The utilization rate of PS in the plateau area was low but did not have any effect upon the mortality rate of neonates with RDS; we suggest that this is related to an insufficient dosage and delayed intervention.

**Figure 4:**
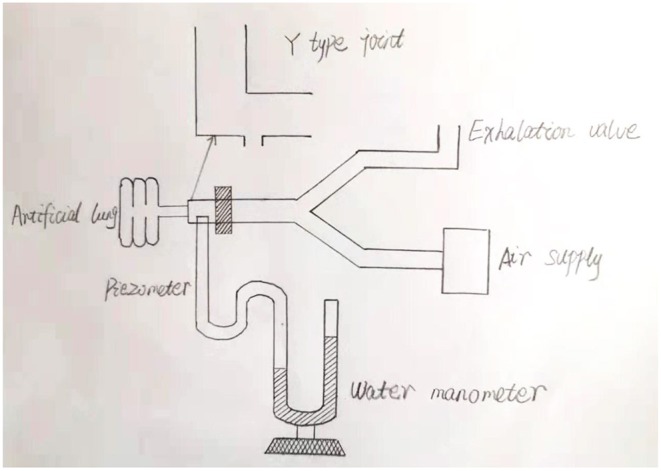
Diagrammatic sketch of water manometer.

To the best of our knowledge, our study, which was carried out in the Naqu area of Tibet, represents the highest elevation described thus far in neonatal research. The highest altitude of neonatal research is 4,340 meters which focus on the incidence of infants born prematurely and the incidence of infants that were smaller than gestational age (13). Thus far, there have been only few studies relating to the application of mechanical ventilation in neonates with RDS at high altitude. Consequently, it is still very unclear as to whether early ventilator parameters should be set differently in high elevations when compared to those used for non-plateau areas. At high altitudes, newborns may have a longer transition period after birth, and more are likely to rely on high concentrations of oxygen and ventilator support during the early stages. We found that the arterial partial pressure of oxygen increased significantly in the high altitude group following mechanical ventilation, thus confirming the positive effect of mechanical ventilation in neonates with RDS at high altitude. The ventilation modes of neonates in the plateau group involved SIMV and only one dose of PS (for economic reasons); so the non-plateau group used the same ventilator and PS conditions. The respiratory frequency was set 40 times per minute in both groups, and the goal of percutaneous oxygen saturation was 90–95%. The first blood gas analysis was carried out after birth and prior to the use of PS and the partial pressure of arterial oxygen in the plateau group was lower than that in the non-plateau group; we consider that the partial pressure of inhaled oxygen in the plateau area was lower than that in the non-plateau area. However, the partial pressure of oxygen in the plateau group at 6 and 12 h after birth was significantly higher than that in the non-plateau group (*p* < 0.001). Due to the relatively poor economy in Naqu, parents need some time to consider whether to use PS or not. In our present study, PS was applied between 5 and 12 h postnatally. Therefore, we consider that the observed significant increase in the arterial partial pressure of oxygen in the plateau group 6–12 h after birth was related to the PS treatment. It may also be related to inappropriate adjustment of ventilator parameters after the application of PS, resulting in an excessive partial pressure of oxygen in the blood, the mean of which exceeded 90 mmHg. There was no significant difference in the partial pressure of carbon dioxide when compared between the two groups but was lower in the non-plateau group at 12 h after birth. We considered that there was a correlation between the partial pressure of carbon dioxide and the reduction of respiratory support after the eventual cessation of invasive ventilation in the non-plateau group. We investigated neonates with severe RDS from both high and low altitudes and found that PIP, PEEP, and FiO2 in the plateau group were significantly higher than those in the non-plateau group (*p* < 0.001). Because the postnatal partial pressure of oxygen and the blood oxygen saturation of newborns in the plateau area are lower than those in the non-plateau area, higher ventilator parameters may be needed to achieve the same levels of oxygen saturation. In addition, previous research has shown that changes in altitude can cause alterations in ventilator function (14–16). The influence of a change in altitude upon different types of ventilators can also differ. Some ventilators provide a lower respiratory frequency, a higher tidal volume, and minute ventilation volume (15, 16), while others do not change significantly (17). This shows that the performance of different ventilators varies when exposed to high altitude. This appears to be related to differences in design features. However, there is no documented evidence of the use of ventilators at high altitudes. Therefore, although the ventilator in the high altitude group in our study was set to 4,500 meters, it is not clear whether the pressure and inhaled oxygen concentration could reach the preset values. As it is found that the pressures in plateau area was obviously higher than that in plain area, we designed a simple water manometer experiment. The ventilator was connected to an artificial lung, and actual pressures were measured by water manometer (see Figure 4). We found when the PIP was set between 20 cm H_2_0 and 30 cm H_2_0, the actual pressure at Y-joint end was about 3 cm H_2_0 lower which indicate that the pressures of ventilators in plateau areas may not reach the preset pressures. However, as this study is a retrospective analysis, it is no longer possible to monitor the pressure of each case in real time. Consequently, it is very important that we understand how to improve ventilator performance at high altitudes. It was also evident in our study that the birth weight in the non-plateau group was lower than in the plateau group; this would also affect settings for PIP and PEEP.

Sub-group analysis showed that the use of PS in the group of newborns surviving in the plateau group was lower than that in the non-plateau group and that the changes of arterial oxygen partial pressure after birth were consistent with those mentioned above. Ventilation in the plateau group required more intensive pressure support and a higher inhaled oxygen concentration. Although there was no significant statistical difference, the duration of mechanical ventilation in the plateau group was longer, this was related to the application of PS and standard ventilator application. When comparing the newborns who survived and those who died in the plateau group, we found that there were no significant differences in terms of gestational age, weight, and PS usage between the two groups, indicating that the use of PS did not reduce the mortality rate for RDS cases; this may have been related to inadequate dosage and an unsatisfactory duration of PS treatment. Postnatal ventilator parameters were significantly higher in the group of RDS patients who died than those who survived. Furthermore, the arterial partial oxygen pressure at 6 and 12 h was lower in those that died when compared to those who survived; this may be related to the greater severity of lung lesions in those that died.

Although the development of mechanical ventilation has greatly reduced the mortality rate associated with neonatal RDS in the Naqu area, our results showed that the mortality rate of cases with severe RDS was still significantly higher than that in the non-plateau area (*P* < 0.01). Generally, once RDS is diagnosed, the earlier PS is used, the better the prognosis (18). If the dose of the first PS administration is 200 mg/kg, then the second dose can be reduced (19). However, the economy of the plateau area is poor and many newborns are not given PS. There are also issues with insufficient dosage and delayed application. Consequently, it is very important to strengthen the use of PS in plateau areas.

Initial mechanical ventilation technology has been developed in Naqu area but the rationality underlying ventilator parameter settings and adjustment urgently requires further evaluation. It is also important to consider that pressure and volume control are the two most commonly used strategies for mechanical ventilation. Volume control can reduce the incidence of bronchopulmonary dysplasia(BPD) and intraventricular hemorrhage and can also shorten the duration of mechanical ventilation (20, 21). Pressure control is currently used during mechanical ventilation in the Naqu area; and the peak inspiratory pressure were high in the plateau group, which probably results in transpulmonary pressure gradients and may lead to injurious lung overdistention, the blood gases also demonstrate a tendency toward hyperventilation, so different types of ventilation strategies are very important in reducing the mortality and adverse outcomes associated with RDS.

## Limitation

There are some limitations to this study which need to be considered. Firstly, there were fewer cases in the plateau group, consequently, there may have been some bias in terms of the statistical analysis. Secondly, the mode of ventilation was relatively fixed. It was therefore not possible to judge the potential significance of different ventilation modes when applied to RDS neonates at high altitude. Most RDS neonates in plain area had changed to non-invasive ventilation mode within 6–12 h, but in Naqu area, the invasive ventilation time was longer. The two groups only compared the parameters of ventilator at birth, and no comparative study was made on the later parameters. Finally, without BPD identification and retinopathy of premature infants(ROP) examination, it was not possible to evaluate secondary damage to the lung and retina caused by the inhalation of hyperbaric oxygen.

## Conclusion

To conclude, our study showed that mechanical ventilation can effectively improve the arterial oxygen partial pressure of newborns with RDS in plateau areas and can reduce the mortality rate associated with this condition. Further research should now be conducted to identify appropriate settings for ventilator parameters and ventilation mode. This will allow us to formulate appropriate guidelines for the mechanical ventilation of newborns with RDS in plateau areas. Creating such guidelines is critical if we are to reduce the neonatal mortality rate in Tibet and improve the mean life expectancy of residents in this area.

## Data Availability Statement

All datasets generated for this study are included in the article/supplementary material.

## Ethics Statement

The studies involving human participants were reviewed and approved by Shengjing Hospital of China Medical University Ethics Committee (Shenyang, China). The study does not contain patient's personal information, fully protects the patient's privacy, has no effect on the routine diagnosis and treatment of patients, patients do not participate in the study for additional tests or examinations. Written informed consent from the participants' legal guardian/next of kin was not required to participate in this study in accordance with the national legislation and the institutional requirements.

## Author Contributions

DC, XL, and JL: study conception, design, analysis, and data interpretation. DC and XL: data acquisition. DC: drafting of the manuscript. JL: critical revision.

### Conflict of Interest

The authors declare that the research was conducted in the absence of any commercial or financial relationships that could be construed as a potential conflict of interest.
